# miR-7704-Enriched Stem Cell-Derived Extracellular Vesicles Attenuate Hyperoxia-Induced Apoptosis and Oxidation in Lung Epithelial Cells

**DOI:** 10.3390/cimb47110893

**Published:** 2025-10-28

**Authors:** Yu-Hsun Chang, Kun-Chi Wu, Dah-Ching Ding

**Affiliations:** 1 Department of Pediatrics, Hualien Tzu Chi Hospital, Buddhist Tzu Chi Medical Foundation, Tzu Chi University, Hualien 970, Taiwan; 2 Department of Orthopedics, Hualien Tzu Chi Hospital, Buddhist Tzu Chi Medical Foundation, Tzu Chi University, Hualien 970, Taiwan; drwukunchi@yahoo.com.tw; 3 Department of Obstetrics and Gynecology, Hualien Tzu Chi Hospital, Buddhist Tzu Chi Medical Foundation, Tzu Chi University, Hualien 970, Taiwan; 4 Institute of Medical Sciences, Tzu Chi University, Hualien 970, Taiwan

**Keywords:** human umbilical cord mesenchymal stem cells, extracellular vesicles, miR-7704, bronchopulmonary dysplasia, hyperoxia-induced apoptosis

## Abstract

Bronchopulmonary dysplasia (BPD) is a significant complication of hyperoxia in preterm neonates. Extracellular vesicle (EV)-based therapies derived from mesenchymal stem cells (MSCs) show regenerative potential. We investigated the therapeutic efficacy of EVs derived from human umbilical cord mesenchymal stem cells (HUCMSCs), particularly those engineered to overexpress miR-7704 in a hyperoxia-induced BPD cell model. EVs were isolated from GFP- and miR-7704-transfected HUCMSCs. A549 alveolar epithelial cells were exposed to normoxic or hyperoxic conditions and treated with HUCMSC-EV or miR-7704-HUCMSC-EV. EV uptake was confirmed using fluorescence microscopy. Cell proliferation was evaluated, and apoptosis was assessed by means of Western blot analysis of caspase family proteins and apoptosis-related markers. Both HUCMSC-EV and miR-7704-HUCMSC-EV enhanced A549 cell proliferation under hyperoxic stress, with miR-7704-HUCMSC-EV showing greater efficacy. Protein-level analyses revealed hyperoxia-induced increases in cleaved caspase-3, caspase-7, and FasL, along with decreased Bcl-2. Treatment with miR-7704-HUCMSC-EV significantly reversed these effects, whereas HUCMSC-EVs minimally impacted apoptotic protein expression. Bioinformatic analysis predicted that hsa-miR-7704 targeted the 3′ UTR of APOPT1. miR-7704-HUCMSC EVs also enhanced the expression of key antioxidant enzymes, including SOD1, SOD2, and HO-1. miR-7704-enriched HUCMSC-derived EV significantly promoted cell survival and mitigated hyperoxia-induced apoptosis and oxidation in a BPD cell model, suggesting their potential therapeutic role in neonatal lung injury.

## 1. Introduction

Bronchopulmonary dysplasia (BPD), defined as oxygen dependence at 36 weeks of postmenstrual age, is among the most common chronic sequelae in premature babies [1]. Surfactant deficiency, oxygen exposure, mechanical ventilation, infection, malnutrition, and patent ductus arteriosus are risk factors for BPD [2]. Oxidative stress is recognized as an important predisposing factor for BPD in premature babies [3].

Currently employed therapies for BPD include respiratory support (e.g., invasive mechanical ventilation, noninvasive pressure support, and oxygen supply), medications (e.g., inhaled bata2 agonists, inhaled or systemic steroids, diuretics, and sildenafil), and nutrition [4]. However, a significant reduction in resident stem cells in the lungs of premature infants with BPD indicates poor regenerative ability to restore normal respiratory function in these children [5]. Stem cell-based therapies, including the transplantation of mesenchymal stem cells (MSCs) and MSC-derived extracellular vesicles (EVs), are promising and effective treatments for BPD [5].

The most common cell models available are immortalized human or animal lung epithelial cells, which have been established using both viral and non-viral immortalization methods to overcome cellular senescence and crises [6]. A549, a human type II alveolar epithelial cell line, was isolated from the lung tissue of a 58-year-old male with lung cancer. This cell line can be used in cancer, immuno-oncology, toxicology, and BPD research [7,8,9].

EVs derived from MSCs show promising therapeutic potential for preventing and treating BPD in preterm infants exposed to hyperoxia. Studies demonstrate that MSC-EVs can ameliorate hyperoxia-induced lung injury, improve alveolarization, and enhance vascular growth in animal models [10,11,12]. The protective effects of EVs are primarily mediated by their bioactive cargo, including vascular endothelial growth factor, which plays a crucial role in attenuating neonatal hyperoxic lung injuries [11]. Intratracheal administration of MSC-EVs has shown superior results compared to MSCs in improving alveolarization and lung vascularization parameters [12]. Additionally, MSC-EVs can suppress the hyperoxia-induced transdifferentiation of alveolar type 2 epithelial cells, possibly through downregulation of WNT5a [10]. These findings suggest that MSC-derived EVs may offer a promising therapeutic approach for the treatment of BPD [13].

MicroRNA-7704 (miR-7704) has been identified as a potential therapeutic target for acute lung injury (ALI) by promoting M2 macrophage polarization through inhibition of the MyD88/STAT1 signaling pathway [14].

In this study, we investigated the therapeutic potential of miR-7704-enriched EVs derived from human umbilical cord mesenchymal stem cells (HUCMSCs) in protecting lung epithelial cells from hyperoxia-induced injury, focusing on their anti-apoptotic and anti-oxidant effects, along with underlying molecular mechanisms.

## 2. Materials and Methods

### 2.1. Ethics

The Research Ethics Committee of Hualien Tzu Chi Hospital (IRB Number: IRB 111-230-B) approved the study protocol.

### 2.2. HUCMSC Culture

HUCMSCs were cultured in low-glucose Dulbecco’s modified Eagle’s medium (DMEM, Gibco, Waltham, MA, USA) supplemented with 10% fetal bovine serum (FBS; Biological Industries, Kibbutz, Israel) and 1% penicillin–streptomycin (Sigma-Aldrich, St. Louis, MO, USA) at 37 °C in a humidified atmosphere with 95% air/5% CO_2_. HUCMSCs were characterized as described previously, using flow cytometry to assess surface markers (CD34, CD44, CD45, CD73, CD90, CD105, HLA-ABC, and HLA-DR; all purchased from BD Biosciences, Franklin Lakes, NJ, USA) and differentiation assays to confirm MSC characteristics [15].

### 2.3. Trilineage Differentiation Capabilities of HUCMSCs

#### 2.3.1. Adipogenesis

HUCMSCs were seeded at a density of 5 × 10^4^ cells per well in a 12-well plate containing adipogenic medium (DMEM supplemented with 10% FBS, 1 μmol/L dexamethasone (Sigma, St. Louis, MO, USA), 5 μg/mL insulin (Sigma), 0.5 mmol/L isobutylmethylxanthine (Sigma), and 60 μmol/L indomethacin (Sigma)). Adipogenesis occurred over 14 days, and the medium was changed every three days. After 14 days, differentiated cells were stained with Oil Red O (Sigma-Aldrich). The cells were harvested for assessing FABP4 and PPARγ gene expression by qRT-PCR.

#### 2.3.2. Osteogenesis

HUCMSCs were seeded at a density of 1 × 10^4^ cells per well in a 12-well plate containing osteogenic medium (DMEM supplemented with 10% FBS, 0.1 μmol/L dexamethasone (Sigma), 10 mmol/L β-glycerol phosphate (Sigma), and 50 μmol/L ascorbate (Sigma)). The medium was changed every three days, and differentiation occurred over 14 days. The differentiated osteoblasts were stained with Alizarin Red (Sigma) after 14 days. The cells were harvested for analyzing ALPL and RUNX2 gene expression by qRT-PCR.

#### 2.3.3. Chondrogenesis

HUCMSCs were seeded at 2.5 × 10^7^ cells/mL on the bottom of a 15-mL conical tube containing 30 μL of chondrogenic medium. The medium comprised DMEM, 10% FBS, 10 ng/mL transforming growth factor-β1 (Pepro Tech, Rocky Hill, NJ, USA), 6.25 μg/mL insulin (Sigma-Aldrich), and 50 μg/mL ascorbic acid-2-phosphate (Sigma-Aldrich). Differentiation occurred over 21 days, following which a globular pellet was formed. Differentiated chondrocytes were cryosectioned and stained with hematoxylin and eosin (H&E), aggrecan, and type II collagen (Sigma). The stained cells were observed under a microscope (Nikon, Tokyo, Japan). The cells were harvested for analyzing the gene expression of ACAN and COL2A1 by qRT-PCR.

### 2.4. qRT-PCR

RNA was extracted using TRIzol (Invitrogen, Waltham, MA, USA) and quantified. Five hundred nanograms of RNA were treated with amplification-grade DNase I (Invitrogen). For qRT-PCR analysis, FastStart Universal SYBR Green Master (ROX, Basel, Switzerland) gene expression assays were performed on the ABI StepOnePlus system (Applied Biosystems, Waltham, MA, USA) with GAPDH as the internal control. The primers and product sizes for the adipogenic, osteogenic, and chondrogenic genes used in the qRT-PCR analysis are listed in Table 1.

### 2.5. Transfection of miR-7704

miR-7704 was encoded into lentivectors and transfected into HUCMSCs following the manufacturer’s instructions (Abm, Vancouver, BC, Canada) and as described previously [16]. Briefly, 1 × 10^5^ HUCMSCs were seeded into a 6-well plate and cultured in DMEM medium containing 10% FBS and 1% penicillin-streptomycin at 37 °C in a humidified atmosphere with 95% air/5% CO_2_. The miR-7704 sequence was CGGGGUCGGCGGCGACGUG. The next day, lentiviral miR-7704 was transfected at an MOI of 2. After 48 h, the virus-containing medium was replaced with a fresh medium. After another 24 h, cells were seeded in a 10 cm dish. Cells were selected using 0.8 μg/mL puromycin for 7 days, following the datasheet information. The remaining cells were cultured in normal medium for two days. The characteristics of miR-7704-HUCMSCs were confirmed using fluorescence microscopy (Zeiss Axio Observer 7, Oberkochen, Germany) and flow cytometry (CD34, CD44, CD45, CD73, CD90, CD105, HLA-ABC, and HLA-DR).

### 2.6. EV Isolation and Identification

EVs were isolated from the conditioned medium (CM) of HUCMSCs, control green fluorescent protein (GFP) HUCMSCs, and miR-7704 HUCMSCs (HUCMSC-Exos, GFP-HUCMSC-Exos, and miR-7704-HUCMSC-Exos). Briefly, 5 × 10^5^ cells were seeded in a 5-layer culture flask (NEST, 731002, Wuxi, China). After 30 h, the cells were washed twice with PBS and cultured in serum-free DMEM (Sigma) supplemented with 1% penicillin-streptomycin (Sigma) for 48 h. The CM was collected and centrifuged at 500× *g* for 5 min to remove cells and cell debris. After centrifugation, a 0.22 μm filter (Sterile Syringe Filter, Sigma) was used to remove the remaining cells and debris from the supernatant. The filtered solution was transferred into a 15 mL Amicon Ultra-15 Centrifugal Filter Unit (MerckMillipore, Burlington, MA, USA) and centrifuged at 4000× *g* for approximately 15–60 min. To recover the concentrated solute, a pipette was inserted into the bottom of the filter device, and the sample was withdrawn using a side-to-side sweeping motion to ensure total recovery. The supernatant was transferred to a sterile vessel, and ExoQuick-TC (SBI, Cat. #EXOTCxxA-1; Palo Alto, CA, USA) was added to the biofluid at a 5:1 ratio. After mixing well by inverting or flicking, the mixture was refrigerated overnight at 4 °C. The following day, the mixture was centrifuged at 1500× *g* for 30 min. The supernatant was aspirated, and the residual ExoQuick-TC solution was centrifuged at 1500× *g* for 5 min. After removing all traces of fluid via aspiration, precipitated EVs formed pellets. The EV-pellet fraction was resuspended in 100–500 μL of EV-Guard EV Storage Buffer 1X (SBI, Cat. EXSBA-1, Palo Alto, CA, USA) for use. EVs were identified using a NanoSight NS300 Instrument (Malvern, Worcestershire, UK), transmission electron microscopy (TEM; HITACHI H-7500, Tokyo, Japan), and a Western blot of CD9 expression (SAB1402143, Sigma-Aldrich, St. Louis, MO, USA).

### 2.7. EV Uptake

The ExoGlow Membrane EV Labeling Kit (SBI, Catalog Number: EXOGM600A-1) was used to determine whether A549 cells could take up EVs. In brief, we added 10 µg of EVs to the labeling reaction buffer, which contained 12 µL of reaction buffer and 2 µL of labeling dye. The samples were mixed and incubated for 30 min at room temperature in the dark. Subsequently, we added 35 µL of ExoQuick-TC to 100 µL of the sample and incubated it overnight at 4 °C. The samples were centrifuged at 10,000 rpm for 10 min. After removing the supernatant, the EV pellet was resuspended in PBS. A549 cells were cultured in the medium until they reached 50–60% confluence. Labeled EVs were added to the A549 cell culture for 24 h. After washing three times with PBS, A549 cells were fixed with 4% paraformaldehyde for 20 min and stained with Hoechst H33342 for 20 min. Images were captured using an Axio Observer 7 fluorescence microscope (Zeiss, Oberkochen, Germany).

### 2.8. Human Alveolar Epithelial Cell Culture and Exposure to Hyperoxia

After thawing in liquid nitrogen, human type II alveolar epithelial A549 cells (ATCC CCL-185, Manassas, VA, USA) were cultured in F-12K Nutrient Mixture (Gibco, Waltham, MA, USA) containing 10% FBS and 1% penicillin/streptomycin for three passages. A549 cells were sub-cultured in F-12K Nutrient Mixture containing 10% EV-depleted FBS and 1% penicillin/streptomycin in 6-well plates at a density of 1 × 10^5^ cells/well. Cells were incubated at 37 °C in a humidified atmosphere with 5% CO_2_ and subjected to either hyperoxia (85% O_2_) for 24, 48, and 72 h or normoxia (21% O_2_) as a control. Hyperoxia experiments were conducted in an incubator chamber (MIC-101; Billirups-Rothenberg Inc., Del-Mar, CA, USA) following the manufacturer’s protocol [17,18].

EV treatment for hyperoxic human alveolar epithelial cells
All cells belonged to one of the following groups:
Control group 1: normoxia
Control group 2: normoxia with HUCMSCs-Exo
Control group 3: normoxia with miR-7704-HUCMSCs-Exo
Study group 1: hyperoxia
Study group 2: hyperoxia with HUCMSCs-Exo
Study group 3: hyperoxia with miR-7704-HUCMSCs-Exo

The EV-to-cell ratio was 500:1 (*v*/*v*). Subsequently, cell proliferation and apoptosis assays were performed to assess the impact of hyperoxia on human alveolar epithelial cells and to assess the therapeutic effects of HUCMSCs-Exo and miR-7704-HUCMSCs-Exo.

### 2.9. Proliferation of Human Alveolar Epithelial Cells

The effects of EVs derived from HUCMSCs (HUCMSC-Exos) and miR-7704-HUCMSCs (miR-7704-HUCMSCs-Exo) on A549 cells were assessed using an XTT cell proliferation kit (2,3-Bis-(2-Methoxy-4-Nitro-5-Sulfophenyl), Biological Industries Ltd., Kibbutz Beit Haemek, Israel) following the manufacturer’s instructions. Briefly, A549 cells were plated in a 96-well microtiter plate at a density of 1.5 × 10^3^ cells per well in a final volume of 100 μL culture medium with/without EVs (7.5 × 10^5^ particles). The cells were incubated with 150 µL of XTT solution for 3 h at 37 °C following the manufacturer’s instructions. Absorbance was measured at 450 nm using a microplate reader (Model 3550, Bio-Rad, Hercules, CA, USA). Growth curves, expressed as optical density values, were constructed at 0, 24, 48, and 72 h for the six groups (three control and three study groups) [16].

### 2.10. Western Blot Analysis

Cells from the six groups were harvested after 72 h and lysed in lysis buffer (150 mM NaCl, 50 mM Tris–HCl, pH 7.4, and 1% Nonidet P-40) containing a proteinase inhibitor cocktail (Roche, Basel, Switzerland). Proteins were separated using 10% sodium dodecyl sulfate-polyacrylamide gel electrophoresis and transferred onto PVDF membranes (Bio-Rad; Immun-Blot PVDF Membranes, cat. no.1620177). Membranes were incubated with specific polyclonal antibodies, followed by incubation with a horseradish peroxidase-conjugated goat anti-rabbit IgG secondary antibody (cat. no.C04003). Bound antibodies were detected using SuperSignal West Pico Plus Chemiluminescent substrate (Thermo Fisher Scientific, Waltham, MA USA). Apoptotic signaling pathway proteins included caspase 3 (Proteintech, cat. no. 19677-1-AP, Rosemont, IL, USA), cleaved-caspase 3 (Cell Signaling, cat. No. #9664, Danvers, MA, USA), caspase 7 (Cell Signaling, cat. No. #12827), cleaved-caspase 7 (Cell Signaling Technology, cat. No. #9491), caspase 8 (Proteintech, cat. No. 66093-1--Ig), cleaved-caspase 8 (Proteintech cat. No. 66093-1--Ig), caspase 9 (ABclonal, cat. No.A11451, Woburn, MA, USA), cleaved-caspase 9 (Abbkine, cat. No. ABP50010, Atlanta, GA, USA), FasL (Abcam, cat. No. G247-4) and BCL2 (Proteintech, cat. No.12789-1-AP). SOD1 (ab308181), SOD2 (ab13533), CAT (ab209211), GPX4 (ab252833), and HO-1 (ab52947) antibodies were purchased from Abcam. β-Actin (Cell Signaling, cat. No.#4970) was used as the control [19].

### 2.11. Bioinformatic Prediction of APOPT1 as an miR-7704 Target

To investigate the potential interaction between hsa-miR-7704 and the APOPT1 gene, bioinformatic analysis was conducted using the miRDB database (http://mirdb.org), a tool for predicting functional microRNA targets based on a support vector machine learning model. The sequence of hsa-miR-7704 was queried against the human transcriptome, with a focus on the 3′ untranslated regions (3′ UTRs) of mRNA targets. APOPT1 (NCBI Gene ID: 84334; GenBank Accession: NM_001302654), a gene involved in regulating mitochondrial apoptosis, was identified as a high-confidence target with a target score of 88.

### 2.12. Statistical Analysis

All data were expressed as the median and range or mean ± standard deviation. Statistical comparisons of the data among the groups were performed using non-parametric tests, such as the Mann–Whitney U test or two-way ANOVA with post hoc analysis with Bonferroni’s test. Differences were considered significant for a *p*-value < 0.05. All statistical analyses were conducted using the SPSS software (version 23, IBM, Armonk, NY, USA).

## 3. Results

### 3.1. Comprehensive Characterization of HUCMSCs and Their Trilineage Differentiation Potential

**Figure 1:**
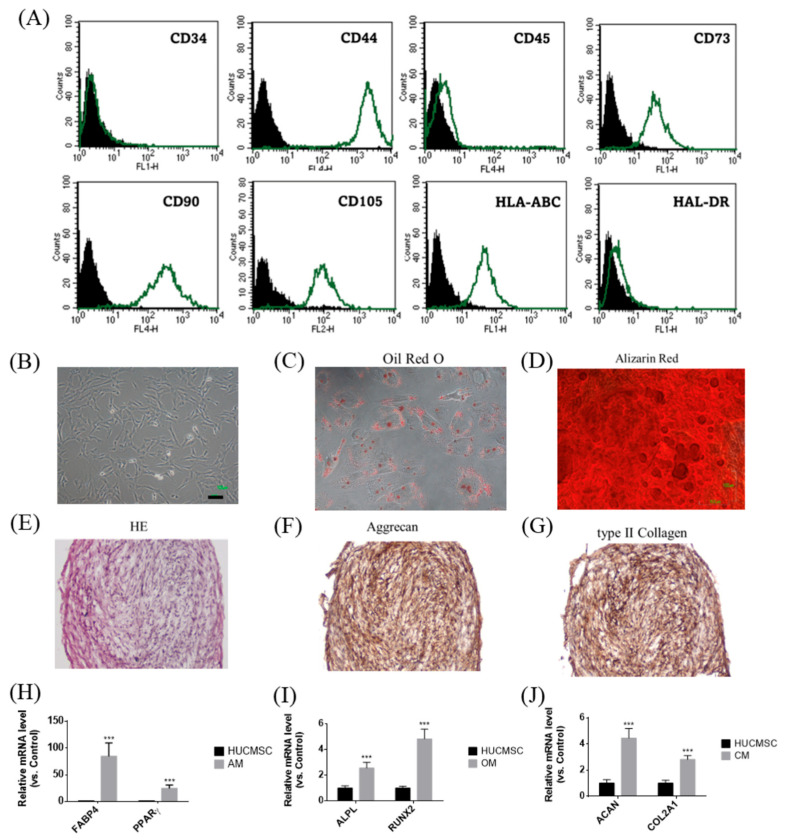
Characteristics of HUCMSCs. (**A**) Flow cytometry analysis of HUCMSCs showed positivity for CD44, CD73, CD90, CD105, and HLA-ABC, and negativity for CD34, CD45, and HLA-DR. Black peak represents the negative control and the green peak indicates antibody-positive cells. (**B**) HUCMSCs exhibiting typical MSC morphology. Scale bar = 100 μm. (**C**) HUCMSCs-derived adipocytes stained positively with Oil Red O. (**D**) HUCMSCs-derived osteocytes stained positively with Alizarin Red. Scale bar = 100 μm. (**E**) H&E staining of HUCMSCs-derived chondrocytes. (**F**) Immunohistochemistry of chondrocytes positive for Aggrecan. (**G**) Immunohistochemistry of chondrocytes positive for type II collagen. (**H**) After adipogenesis, cells (AM) showed positive expression of FABP4 and PPARγ genes. (**I**) After osteogenesis, cells (OM) showed positive expression of ALPL and RUNX2 genes. (**J**) After chondrogenesis, cells (CM) showed positive expression of ACAN and COL2A1 genes. *** *p* < 0.001 compared with the control.

Figure 1 illustrates the phenotypic and functional characteristics of HUCMSCs. Flow cytometry analysis confirmed the expression of mesenchymal markers (CD44, CD73, CD90, CD105, and HLA-ABC) but not hematopoietic and immunogenic markers (CD34, CD45, and HLA-DR) in HUCMSCs (Figure 1A). Morphologically, cells displayed the typical spindle-shaped MSC appearance (Figure 1B). Their multipotent differentiation capabilities have been demonstrated by the successful induction of adipogenesis, osteogenesis, and chondrogenesis. Adipocytes stained positively for Oil Red O (Figure 1C) and expressed FABP4 and PPARγ (Figure 1H); osteocytes were stained with Alizarin Red (Figure 1D) and expressed ALPL and RUNX2 (Figure 1I); and chondrocytes were identified by H&E staining (Figure 1E), immunohistochemical positivity for Aggrecan (Figure 1F) and type II collagen (Figure 1G), and gene expression of ACAN and COL2A1 (Figure 1J). In summary, HUCMSCs exhibited defined MSC surface markers and robust trilineage differentiation capacity, confirming their potential for regenerative and therapeutic applications.

### 3.2. Phenotypic Stability of HUCMSCs Following Lentiviral Transfection

**Figure 2:**
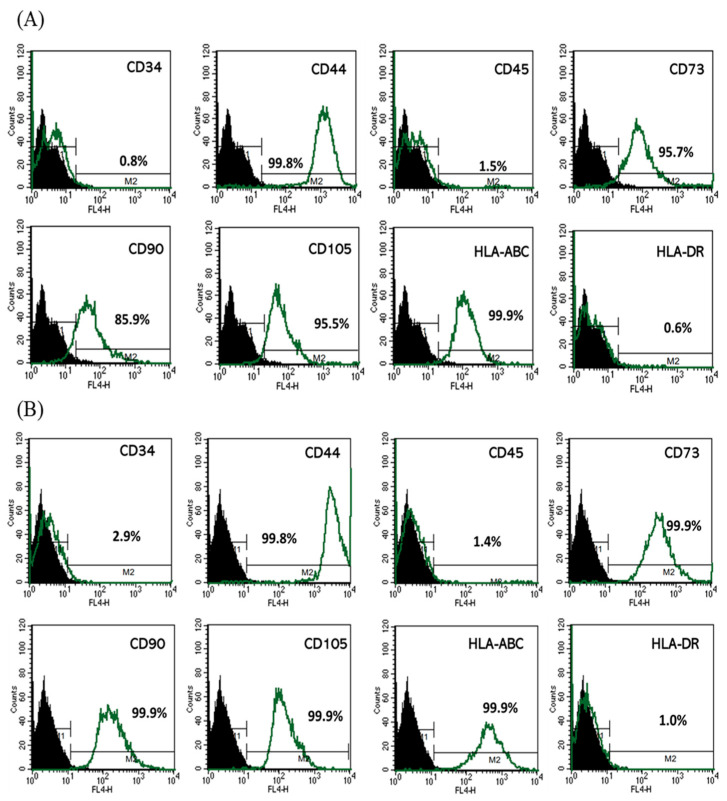
Characteristics of HUCMSCs after Lentiviral Transfection. Flow cytometry analysis of (**A**) GFP-transfected HUCMSCs and (**B**) miR-7704-transfected HUCMSCs, showing positivity for CD44, CD73, CD90, CD105, and HLA-ABC, and were negative for CD34, CD45, and HLA-DR. Black peak represents the negative control and the green peak indicates antibody-positive cells.

Figure 2 shows that lentiviral transfection with either GFP or miR-7704 did not alter the mesenchymal stem cell phenotype of HUCMSCs. Flow cytometric analysis showed that both GFP-transfected (A) and miR-7704-transfected (B) HUCMSCs retained the expression of key MSC surface markers (CD44, CD73, CD90, CD105, and HLA-ABC) and remained negative for hematopoietic and immunogenic markers (CD34, CD45, and HLA-DR), indicating preserved cellular identity and stemness post-transduction.

### 3.3. Characterization of miR-7704-Transfected HUCMSCs and Their Derived EVs

**Figure 3:**
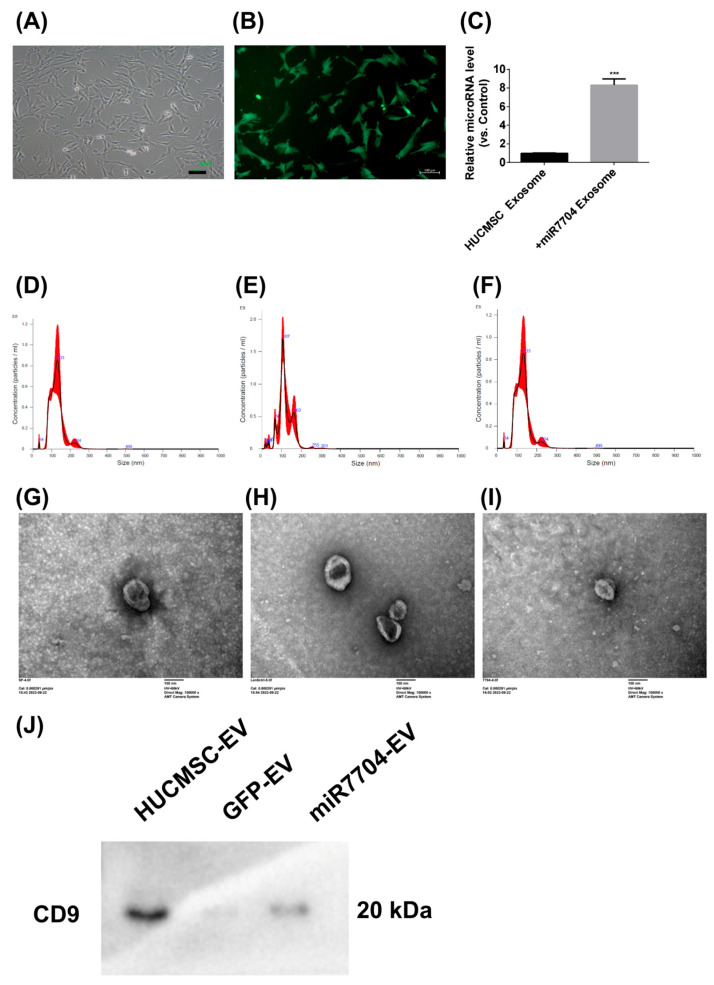
Characteristics of Lentiviral miR-7704-Transfected HUCMSCs and Derived EVs. (**A**) miR-7704-transfected HUCMSCs exhibited typical MSC morphology. Scale bar = 100 μm. (**B**) miR-7704-HUCMSCs expressing GFP. Scale bar = 100 μm. (**C**) miR-7704-HUCMSCs-derived EVs show significantly higher miR-7704 expression. *** *p* < 0.001 compared with the control. (**D**) The mean size and concentration of HUCMSCs-derived EVs were 133.5 nm and 7.36 × 10^9^ particles/mL, respectively. (**E**) The mean size and concentration of control GFP HUCMSCs-derived EVs were 120.1 nm and 7.88 × 10^9^ particles/mL, respectively. (**F**) The mean size and concentration of miR-7704-HUCMSCs-derived EVs were 128.3 nm and 5.7 × 10^9^ particles/mL, respectively. (**G**–**I**) Electron microscopy images of HUCMSCs, control GFP HUCMSCs, and miR-7704-HUCMSC-derived EVs. Scale bar = 100 nm. (**J**) Western blot analysis of CD9 expression in HUCMSCs, GFP-transfected HUCMSCs, and miR-7704-HUCMSC-derived EVs.

Figure 3 shows the morphological and functional characteristics of miR-7704-transfected HUCMSCs and their EVs. Transfected HUCMSCs retained typical MSC morphology (Figure 3A) and expressed GFP (Figure 3B). EVs derived from these cells exhibited significantly elevated miR-7704 levels (Figure 3C), confirming successful genetic modifications. Nanoparticle tracking analysis showed comparable size distributions among EVs from unmodified (133.5 nm), GFP-transfected (120.1 nm), and miR-7704-transfected (128.3 nm) HUCMSCs, with slight variations in particle concentration (Figure 3D–F). Transmission electron microscopy confirmed the typical cup-shaped morphology of EVs from all groups (Figure 3G–I), verifying their successful isolation and structural integrity. CD9 expression was detected in all three groups—HUCMSCs, GFP-transfected HUCMSCs, and miR-7704-HUCMSC-derived EVs (Figure 3J). In summary, miR-7704-transfected HUCMSCs maintained MSC morphology and successfully produced EVs with elevated miR-7704 expression and typical size and structure.

### 3.4. Visualization of EV Uptake by A549 Cells

**Figure 4:**
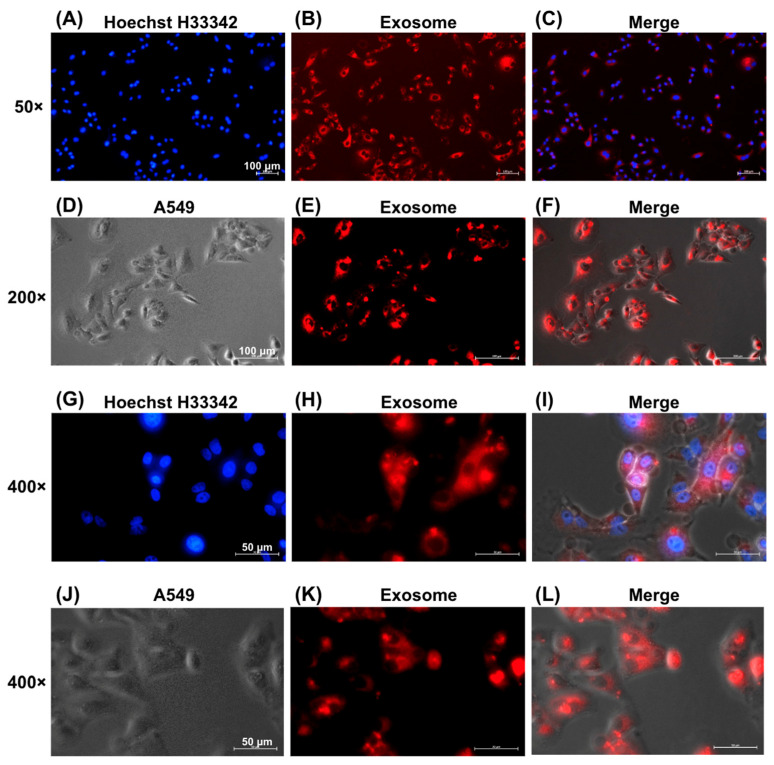
Uptake of EVs by A549 Cells. EVs were labeled with red fluorescence using the ExoGlowTM-Membrane EV Labeling Kit. Fluorescence microscopy images showing uptake of red-labeled exosomes by A549 cells, with nuclei stained blue by Hoechst H33342 and merged images indicating intracellular localization. Magnification: (**A**–**C**) 50×, scale bar = 100 μm, (**D**–**F**) 200×, scale bar = 100 μm, and (**G**–**L**) 400×, scale bar = 50 μm.

Figure 4 shows the successful internalization of EVs by A549 lung cancer cells, as evidenced by fluorescence microscopy. EVs labeled with red fluorescence via the ExoGlow™-Membrane EV Labeling Kit were observed within the cytoplasm of A549 cells at increasing magnifications: 50× (Figure 4A–C), 200× (Figure 4D–F), and 400× (Figure 4G–J). Red fluorescence confirmed effective uptake of labeled EVs by recipient cells.

### 3.5. HUCMSC-EVs and miR-7704-Enriched EVs Promote A549 Cell Growth Under Hyperoxic Conditions

**Figure 5:**
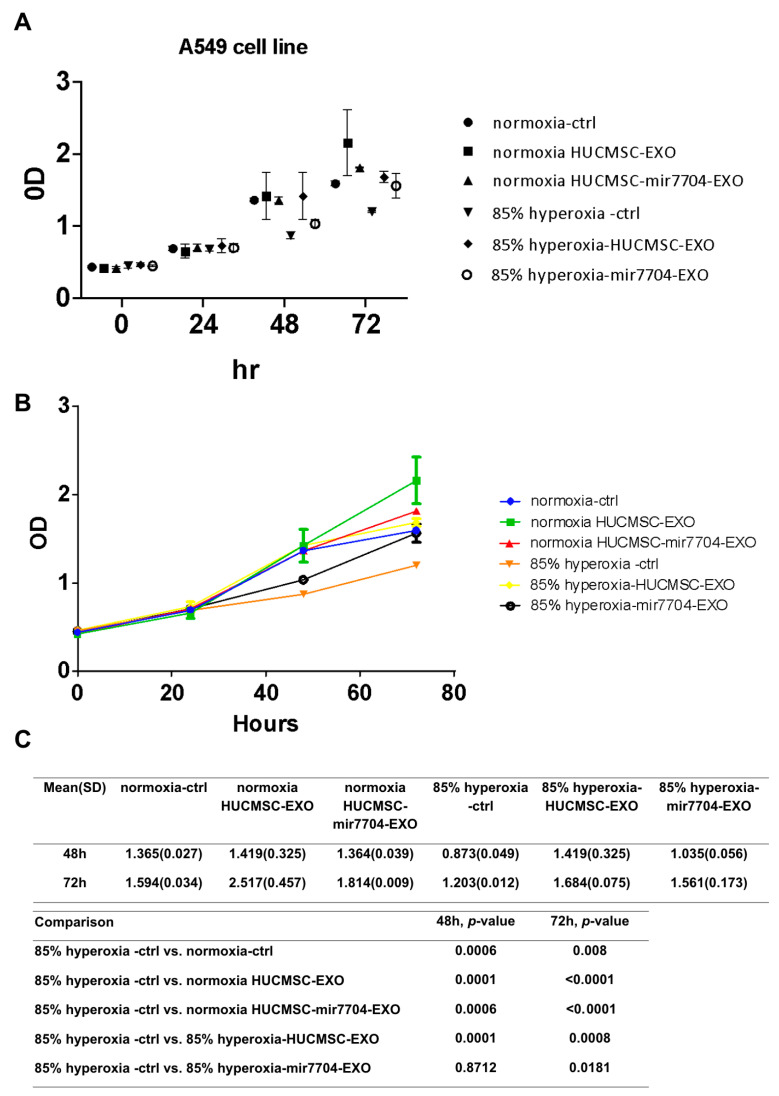
Effects of miR-7704-enriched EVs on A549 cell proliferation under hyperoxic conditions. (**A**) Cell viability of A549 cells cultured under normoxia or 85% hyperoxia for 0–72 h, treated with control medium, HUCMSC-derived EVs, or miR-7704-enriched HUCMSC EVs, was measured by XTT assay. (**B**) Growth curves indicate that hyperoxia significantly suppresses cell proliferation, whereas treatment with miR-7704-EVs partially restores cell viability compared to the hyperoxia control group. (**C**) A statistical comparison of OD values at 48 and 72 h revealed significant differences between the hyperoxia control and normoxia groups, as well as between the hyperoxia control and HUCMSC-EV-treated groups. In contrast, miR-7704-EV treatment showed attenuated hyperoxia-induced growth inhibition. Data are presented as the mean ± standard deviation (SD) (*n* = 3); *p* < 0.05 was considered statistically significant.

Under hyperoxic conditions, A549 cell proliferation was markedly reduced compared to normoxic controls, confirming the cytotoxic effects of oxidative stress. Treatment with HUCMSC-derived EVs significantly improved cell viability, and this effect was further enhanced by miR-7704-enriched EVs. As shown in Figure 5, XTT assays revealed that miR-7704-EVs partially restored cell proliferation at both 48 and 72 h compared with the hyperoxia control group (*p* < 0.05). These findings indicate that miR-7704-EVs can alleviate hyperoxia-induced growth inhibition, suggesting their protective role against oxidative stress-mediated cellular injury.

### 3.6. miR-7704-Enriched EVs Attenuate Apoptosis Marker Expression in Hyperoxia-Exposed A549 Cells

**Figure 6:**
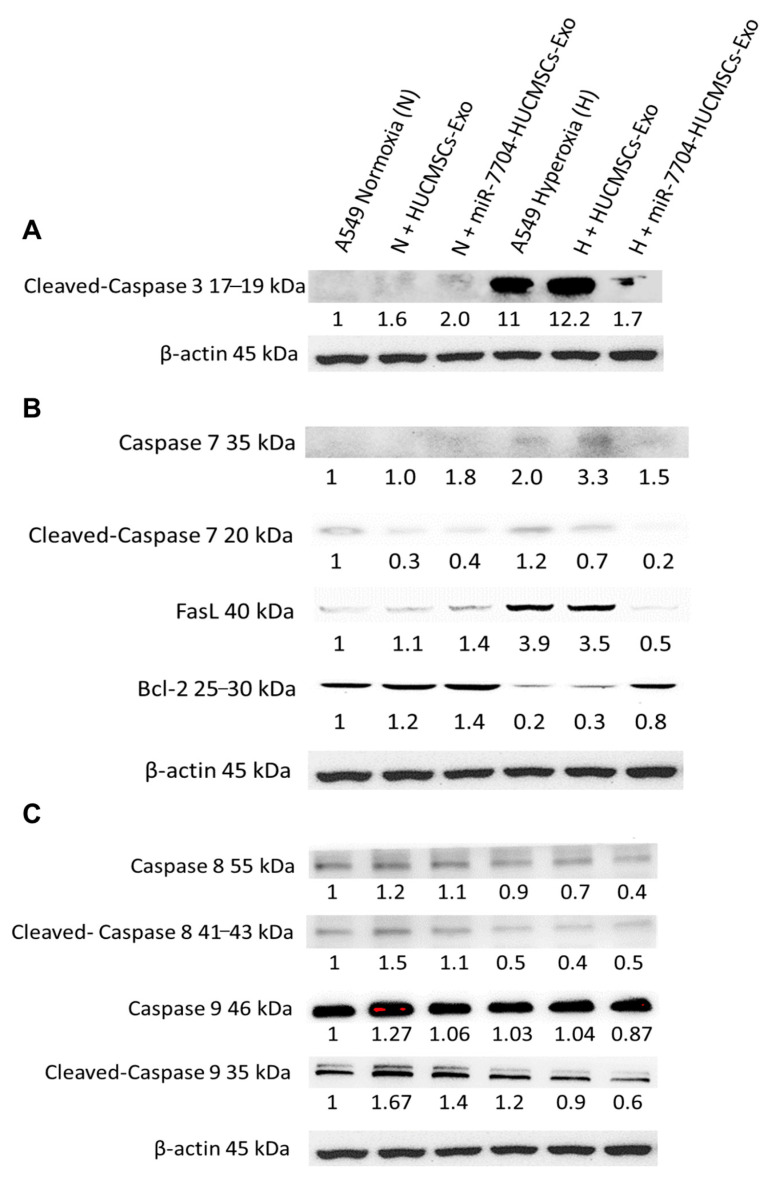
Western Blotting of Apoptosis Pathway after EV Therapy in BPD Model. (**A**) Cleaved-caspase 3 levels were significantly higher in the hyperoxia group compared to the normoxia group. After miR-7704-HUCMSCs-Exo treatment, cleaved-caspase 3 levels decreased significantly. No significant change in the level of cleaved-caspase 3 was observed after HUCMSC-Exo treatment. (**B**) Caspase 7 and FasL levels were significantly higher in the hyperoxia group compared to the normoxia group, while Bcl-2 levels were significantly lower. After miR-7704-HUCMSC-Exo treatment, cleaved-caspase 7 and FasL levels decreased significantly, while Bcl-2 levels increased significantly. No significant changes in FasL and Bcl-2 levels were observed after HUCMSC-Exo treatment. (**C**) No significant changes were observed in caspase 8 and caspase 9 levels in A549 cells under hyperoxic conditions.

Figure 6 shows the Western blot analysis of apoptosis-related proteins in A549 cells under hyperoxic stress and following EV treatment. In the hyperoxic group, cleaved-caspase 3, caspase 7, and FasL levels were significantly elevated, whereas anti-apoptotic Bcl-2 levels were decreased, indicating enhanced apoptosis. Treatment with miR-7704-HUCMSC-derived EVs significantly reduced cleaved-caspase 3, caspase 7, and FasL levels, and increased Bcl-2 expression, demonstrating a protective anti-apoptotic effect. In contrast, treatment with HUCMSC-Exos alone did not significantly alter the levels of cleaved caspase 3, FasL, or Bcl-2. No significant changes in caspase 8 or caspase 9 levels were observed under hyperoxic conditions, suggesting their limited involvement in this model.

### 3.7. Predicted Binding of miR-7704 to APOPT1 Suggests Post-Transcriptional Regulation of Mitochondrial Apoptosis

**Figure 7:**
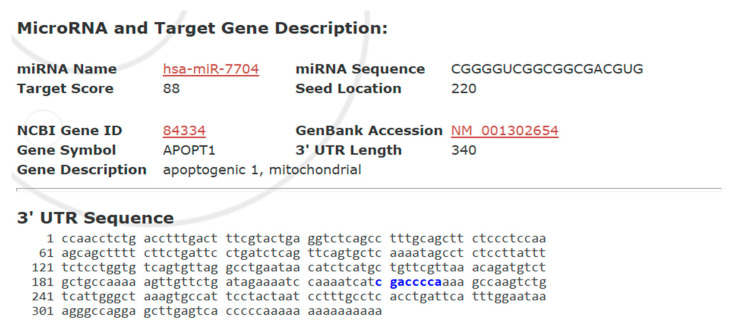
Predicted Target Interaction Between hsa-miR-7704 and APOPT1 3′ UTR. Predicted binding site of hsa-miR-7704 within the 3′ untranslated region (3′UTR) of the APOPT1 gene, where the blue-highlighted sequence (gaccca) indicates the complementary region targeted by the miR-7704 seed sequence.

Figure 7 presents a bioinformatic prediction of the interaction between hsa-miR-7704 and the 3′ untranslated region (UTR) of the APOPT1 gene, encoding a mitochondrial apoptosis-related protein. With a high target score of 88, miR-7704 was predicted to bind at position 220 of the 340-nucleotide 3′ UTR of APOPT1, suggesting a strong likelihood of post-transcriptional regulation. The identified seed region-binding site supports the hypothesis that miR-7704 suppresses APOPT1 expression, thereby modulating mitochondrial apoptosis signaling.

Bioinformatics analysis identified APOPT1 (apoptogenic 1, mitochondrial) as a putative target gene of hsa-miR-7704, with a high target score of 88. The miRNA seed sequence (CGGGGUC) was predicted to bind at position 220 of the 3′ untranslated region (UTR) of APOPT1 (NCBI Gene ID: 84334, GenBank Accession: NM_001302654), which is 340 nucleotides in length. The complementary binding region within the 3′ UTR is highlighted in blue. This suggests that miR-7704 may post-transcriptionally regulate APOPT1, potentially modulating mitochondrial apoptotic pathways.

### 3.8. miR-7704 EVs Enhance Antioxidant Enzyme Expression Under Hyperoxic Conditions

**Figure 8:**
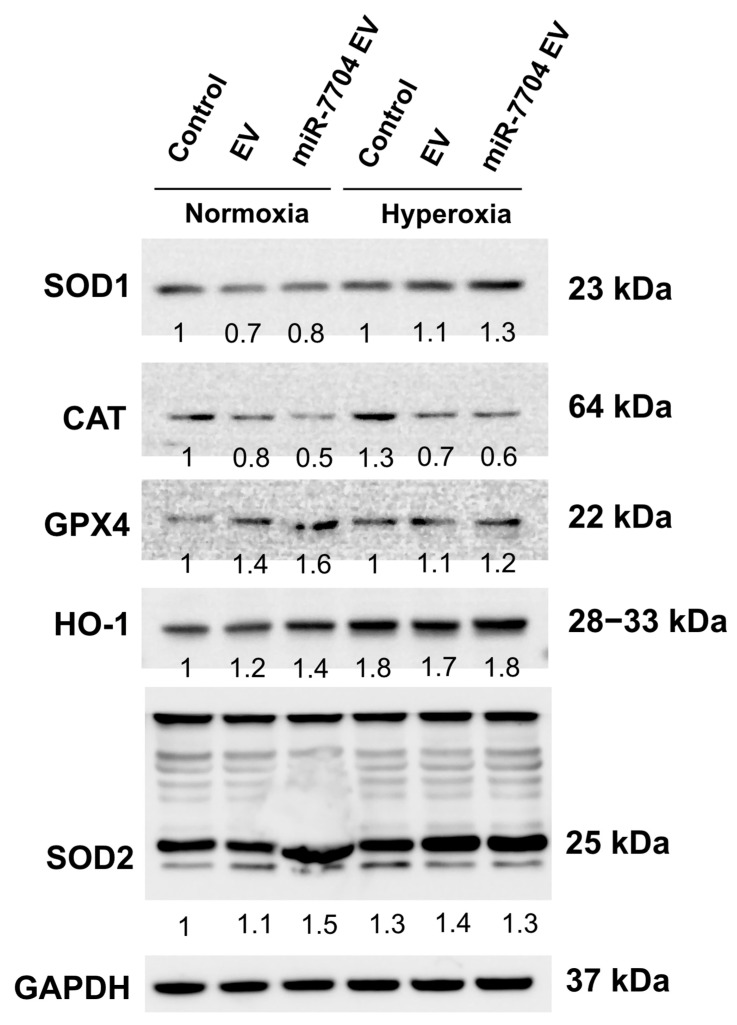
miR-7704-EV treatment enhances antioxidant response under hyperoxic conditions. Representative Western blot analysis of antioxidant enzymes and stress-related proteins, including SOD1, CAT, GPX4, HO-1, and SOD2, in control, EV-treated, and miR-7704 EV-treated cells cultured under normoxia or hyperoxia. GAPDH served as the loading control. Densitometric values normalized to GAPDH are shown below each band, with the control under normoxia set as 1. Under hyperoxic stress, miR-7704 EV increased the expression of SOD1, HO-1, and SOD2 compared with the control and EV groups, indicating an enhanced antioxidant defense capacity.

Under hyperoxic stress, the expression of antioxidant and stress-related proteins was differentially modulated by miR-7704-enriched EV. Western blot analysis revealed that hyperoxia slightly increased the levels of SOD1, HO-1, and SOD2 in both control and EV-treated groups, whereas CAT expression declined (Figure 8). Notably, miR-7704 EV treatment further enhanced the expression of SOD1, HO-1, and SOD2 compared to EVs alone, indicating potentiation of the antioxidant defense system. GPX4 expression remained relatively stable across conditions, suggesting selective upregulation of specific oxidative stress regulators. These findings suggest that miR-7704 EVs enhance the endogenous antioxidant response under hyperoxic conditions, potentially mitigating oxidative stress and injury.

## 4. Discussion

This is the first study to develop transgenic miR-7704-HUCMSCs that substantially secrete substantial amounts of miR-7704-enriched EVs. These EVs effectively reduced apoptosis and stimulated cell proliferation after hyperoxia treatment in a BPD cell model. HUCMSCs transfected with miR-7704 provide a therapeutic strategy for BPD by enriching miR-7704 in HUCMSC EVs. HUCMSC EVs also stimulate cell proliferation.

BPD is characterized by impaired alveolarization, chronic oxidative stress, and dysregulated cell survival in the immature lung [20]. In this context, our finding that miR-7704 attenuates hyperoxia-induced apoptosis in A549 cells provides a potential mechanistic link to the pathophysiology of BPD. By modulating APOPT1, a mitochondrial protein required for cytochrome c oxidase activity, miR-7704 may help preserve mitochondrial integrity and reduce epithelial cell loss under oxidative stress [21]. These results suggest that miR-7704-mediated regulation of mitochondrial apoptosis pathways may contribute to protecting alveolar epithelial cells, thereby offering new insights into the molecular processes underlying BPD.

miR-7704 has been implicated in various diseases. Lin et al. demonstrated that miR-7704 promoted M2 macrophage polarization by inhibiting the MyD88/STAT1 signaling pathway, thereby improving pulmonary function and survival in models of acute lung injury [14]. Wu et al. showed that EVs from HUCMSCs transfected with miR-7704 improved walking capacity, preserved cartilage morphology, and reduced matrix metalloproteinase 13 (MMP13) expression in a mouse model of osteoarthritis [22]. miR-7704 shows broad therapeutic potential by modulating inflammation, promoting tissue repair, and protecting against oxidative damage in various disease models. In our study, miR-7704-HUCMSC-EV showed anti-apoptotic activity in lung epithelial cells.

Chang et al. performed intratracheal transplantation of human umbilical cord blood-derived MSCs into neonatal rats and found it more effective than intraperitoneal transplantation in attenuating hyperoxia-induced lung injury in neonatal rats [23]. Additionally, Zhou et al. showed that DiI-labeled human breast milk-derived EVs targeted lung tissue in neonatal rats 12 h after intragastric gavage administration [18]. In this study, we successfully used an EV-labeled dye to identify EVs incorporated into cells after hyperoxia. After EV transplantation, the cells exhibited anti-apoptotic capabilities under hyperoxic culture conditions.

In this study, A549 cells were used to mimic lung epithelial cell changes under hyperoxic stress. While A549 is a human alveolar epithelial cell line, it is frequently applied in BPD research as an accessible in vitro model [7,8,9]. Compared with animal models that better replicate the developmental and multicellular complexity of neonatal lungs, A549 cells provide a reproducible and cost-effective system for investigating molecular mechanisms [7,8,9], although their limitations in fully reflecting in vivo pathophysiology should be acknowledged.

Although A549 cells cannot fully recapitulate the developmental and multicellular complexity of the neonatal lung, this model provides a reproducible and human-derived system to study hyperoxia-induced injury. The observed protective effect of miR-7704 suggests that such an in vitro platform can be leveraged to screen candidate molecules or test interventions that modulate oxidative stress and apoptosis. Therefore, while translational validation in animal models remains essential, our findings indicate that A549-based assays may serve as a useful preliminary tool for identifying potential therapeutic strategies to prevent or treat BPD.

BPD-related mechanisms of hyperoxia-induced injury involve multiple apoptotic pathways. Endoplasmic reticulum stress-associated apoptosis via the IRE1α pathway has been rarely reported [24]. In mouse alveolar epithelial cells, hyperoxia-induced apoptosis was mediated through the IL-17 pathway [25]. In contrast, in MLE-12 lung epithelial cells, hyperoxia triggered Fas/FasL expression and apoptosis [26]. Emerging evidence also highlights the role of microRNA regulation, such as miR-3202, which inhibits BPD-associated apoptosis and oxidative stress in bronchial epithelial cells by targeting RAG1 [27]. Additionally, hyperoxia has been shown to induce both ferroptosis and apoptosis by downregulating PLAGL2 and suppressing the HIF-1α/VEGF signaling pathway in neonatal alveolar type II epithelial cells [27]. In our study, we found that APOPT1 may be implicated in the anti-apoptotic effect of miR-7704 on A549 cells under hyperoxic conditions. APOPT1 encodes a mitochondrial protein essential for cytochrome c oxidase (COX) function, suggesting a potential link between mitochondrial homeostasis and miR-7704-mediated cytoprotection.

Our findings demonstrated that miR-7704 EVs enhanced the expression of key antioxidant enzymes, including SOD1, SOD2, and HO-1, under hyperoxic conditions, indicating activation of endogenous antioxidant defense pathways. This result suggests that miR-7704 EVs can mitigate oxidative stress, which is a major contributor to hyperoxia-induced alveolar injury and BPD. Previous studies have demonstrated that oxidative stress leads to mitochondrial dysfunction and apoptosis in developing lungs, and that antioxidant enzymes, such as SOD2 and HO-1, play crucial roles in protecting neonatal alveolar epithelial cells from hyperoxia-induced injury [28,29]. Consistent with these reports, our data indicate that miR-7704 EVs may exert their protective effect by augmenting cellular antioxidant capacity and attenuating oxidative damage. Similar antioxidant effects have also been observed with mesenchymal stem cell-derived EVs in models of lung injury, which were shown to reduce reactive oxygen species and enhance expression of antioxidative genes [30]. Together, these findings support the potential therapeutic role of miR-7704 EVs in alleviating oxidative stress-induced injury in hyperoxic lung environments and provide mechanistic insight into their possible application for preventing or treating BPD.

### Strengths and Limitations

This study presents a comprehensive and methodologically sound approach to characterize both unmodified and genetically engineered HUCMSCs, validating their identity, multipotency, and post-transfection stability. The successful delivery of miR-7704 via EVs and its functional effects in an oxidative injury model (A549 cells under hyperoxia) provided compelling evidence for the therapeutic utility of engineered EVs. The integration of molecular, cellular, and bioinformatic analyses strengthens the biological relevance of these findings, particularly the demonstration of reduced apoptosis and enhanced cell viability in response to miR-7704-enriched EVs. Moreover, the use of predictive miRNA target analysis to identify APOPT1 as a potential downstream effector will add mechanistic depth and direction for future studies.

Nevertheless, this study was limited by the use of a single cell line (A549) to model oxidative lung injury, which may not fully recapitulate the complexity of bronchopulmonary dysplasia or the in vivo responses. Future validation using primary neonatal alveolar epithelial cells or animal models of hyperoxia-induced bronchopulmonary dysplasia is warranted to confirm the translational relevance of our findings. Additionally, the biodistribution, immune clearance, off-target effects, and safety of miR-7704 EVs in suitable animal models will be assessed to evaluate their therapeutic feasibility. Functional validation of the miR-7704-APOPT1 interaction was limited to in silico prediction, and no direct assays (e.g., luciferase reporter or protein-level analysis of APOPT1) were performed to confirm this regulatory relationship. A limitation of this study is that only GFP-transfected HUCMSCs were used as controls, without inclusion of mock- or non-targeting miRNA vector-transfected cells, which may not fully exclude the potential cellular stress effects induced by lentiviral transduction.

## 5. Conclusions

We comprehensively characterized HUCMSCs and confirmed their mesenchymal identity, as well as their robust trilineage differentiation capacity. Importantly, lentiviral transfection with miR-7704 did not alter the stem cell properties, allowing for the stable generation of miR-7704-enriched EVs. These EVs were efficiently taken up by A549 lung epithelial cells and demonstrated a significant capacity to promote cell growth and attenuate apoptosis under hyperoxic stress, as evidenced by increased cell proliferation, reduced cleaved caspase levels, and restored Bcl-2 expression. Additionally, bioinformatics analysis suggested that miR-7704 might target APOPT1, implicating a potential role in regulating mitochondrial apoptosis pathways. miR-7704-HUCMSC EVs also enhanced the expression of key antioxidant enzymes, including SOD1, SOD2, and HO-1.

The findings highlight the therapeutic potential of miR-7704-enriched HUCMSC-derived EVs in protecting epithelial cells from oxidative stress-induced apoptosis and oxidation, such as in BPD. The ability to engineer stem cell-derived EVs with specific miRNA cargos opens up new avenues for precise EV therapy for pulmonary diseases. Moreover, the predicted targeting of APOPT1 by miR-7704 introduces a novel regulatory mechanism that merits further investigation of mitochondrial apoptosis modulation and the broader applications of miR-7704-based interventions.

## Figures and Tables

**Table 1:** Primers and product sizes of adipogenic, osteogenic, and chondrogenic genes were analyzed by means of qRT-PCR.

Gene	Forward	Reverse	Product Size (bp)
*FABP4*	ATGGGATGGAAAATCAACCA	GTGGAAGTGACGCCTTTCAT	87
*PPARγ*	CCAGAAAGCGATTCCTTCAC	TGCAACCACTGGATCTGTTC	240
*RUNX2*	CGGAATGCCTCTGCTGTTAT	TTCCCGAGGTCCATCTACTG	174
*ALPL*	CCACGTCTTCACATTTGGTG	GCAGTGAAGGGCTTCTTGTC	96
*ACAN*	CGAAACATCACTGAGGGTGA	GCAAACGTGAAGGGCTCCT	107
*COL2A1*	GAGAGGTCTTCCTGGCAAAG	AAGTCCCTGGAAGCCAGAT	118
*GAPDH*	GGTCTCCTCTGACTTGAACA	GTGAGGGTCTCTCTCTTCCT	221

## Data Availability

The original contributions presented in this study are included in the article. Further inquiries can be directed to the corresponding authors.
